# The Malaria Parasite

**Published:** 1900-05-19

**Authors:** 


### THE MALARIA PARASITE.

At the last meeting of the Balneological and Climatological Society, during a discussion on the climate of Rhodesia, some remarks of considerable interest were made by Dr. Alfred Hillier in regard to a point in the natural history of malaria, to which we have more than once directed attention, namely, the probability that man is not the only host of the malaria parasite from whom the mosquito can become infected. That the parasite is found in certain species of mosquito, and that it is conveyed by them to men, no reasonable student of the question can have any doubt; but when it is held, as seems to be the case by some, that the parasite can only be obtained by the mosquito from man, and can only be conveyed by the mosquito to man, Dr. Hillier thinks, and we think, that the theory goes beyond the proof, and he points to certain facts which appear to militate against it. For instance, as he says, Rhodesia is a huge country of 150,000 square miles, while its entire population is estimated at 300,000 blacks and 15,000 white. Very large portions of the country, therefore, are uninhabited, and in some of these districts — the low-lying, swampy ground, where malaria is most virulent—there is in many instances no population at all, yet parties of hunters wandering in such districts are often prostrated to a man by malaria. This being so, Dr. Hillier very properly asks, From whom can the mosquitos in these cases have derived the parasite? and confesses to considerable doubt as to whether man is the only host from whom the parasite may be derived. The question is, of course, one of the greatest possible interest on account of its practical bearing upon the measures to be taken for the prevention of the disease.

